# Supplementary material for: Evaluation of Viral Suppression in Paediatric Populations: Implications for the Transition to Dolutegravir-Based Regimens in Cameroon: The CIPHER-ADOLA Study
Source: Biomedicines. 2024 Sep 12;12(9):2083. doi: 10.3390/biomedicines12092083 (PMC11440115; doi:10.3390/biomedicines12092083)
Supplement: Supplementary file 1 [file biomedicines-12-02083-s001.zip › biomedicines-3105317-supplementary.pdf]

biomedicines

MDPI

Supplementary Table S1. Factors associated with virological suppression among children.

Factors	Regression model			
	Crude OR (95% CI)	p-value	Adjusted OR (95% CI)	p-value
**Sex**				
Males	0.791 (0.598-1.048)	0.102		
Females	1			
**Age categories, years**				
**<5**	**0.642 (0.482-0.856)**	**0.003**	**0.596 (0.433-0.819)**	**0.001**
5-9	1		1	
**Treatment duration, month**				
6	0.967 (0.641-1.458)	0.871	1.244 (0.800-1.934)	0.332
12	0.775 (0.523-1.147)	0.203	0.959 (0.634-1.451)	0.842
24	1.104 (0.720-1.692)	0.651	1.375 (0.876-2.157)	0.167
36	0.517 (0.294-0.911)	0.022	0.584 (0.329-1.037)	0.066
>36	1		1	
ART line				
1st line	1			
2nd line	0.758 (0.570-1.008)	0.057		
**Backbone**				
TDF/3TC	1		1	
ABC/3TC	0.753 (0.464-1.222)	0.251	0.677 (0.411-1.113)	0.124
AZT/3TC	0.909 (0.381-2.169)	0.830	0.809 (0.334-1.959)	0.639
**Anchor drug**				
DTG	1			
EFV/NVP	0.734 (0.466-1.158)	0.184		
ATV/r/LPV/r	0.626 (0.417-0.941)	0.024		

3TC: Lamivudine; ABC: abacavir; ART: antiretroviral treatment; ATV: atazanavir; AZT: zidovudine; DTG: Dolutegravir; EFV: efavirenz; LPV: lopinavir; NVP: nevirapine; TDF: tenofovir disoproxil fumarate; OR: odds ratio; TDF: tenofovir disoproxil fumarate. CI: confidence interval. ART: antiretroviral therapy. For the multivariate analysis, the model was adjusted for age, treatment duration, and NRTI backbone. P-values in boldface indicates those that were significantly associated ($p < 0.05$) with virological suppression.

Supplementary Table S2. Factors associated with virological suppression among adolescents.

Factors	Regression model			
	Crude OR (95% CI)	p-value	Adjusted OR (95% CI)	p-value
**Sex**				
Males	0.847 (0.688-1.042)	0.117		
Females	1			
**Age categories, years**				
5-9	0.873 (0.712-1.071)	0.192	0.978 (0.787-1.216)	0.844
10-14	1		1	
**Treatment duration, month**				
6	0.977 (0.727-1.312)	0.877		
12	1.251 (0.878-1.784)	0.216		
24	1.080 (0.762-1.529)	0.666		
36	0.861 (0.509-1.457)	0.577		
>36	1		1	
**ART line**				
1st line	1			
2nd line	0.442 (0.317-0.615)	<0.001		
3rd line	0.112 (0.010-1.242)	0.075		
**Backbone**				
TDF/3TC	1		1	
ABC/3TC	**0.474 (0.338-0664)**	**<0.001**	**0.610 (0.419-0.889)**	**0.010**
AZT/3TC	0.419 (0.230-0.762)	0.004	0.670 (0.351-1.279)	0.225
**Anchor drug**				
DTG	1			
EFV/NVP	**0.698 (0.525-0.929)**	**0.014**	**0.746 (0.557-0.999)**	**0.049**
ATV/r/LPV/r/DRV/r	**0.385 (0.276-0.538)**	**<0.001**	**0.477 (0.329-0.693)**	**<0.001**

3TC: Lamivudine; ABC: abacavir; ART: antiretroviral treatment; ATV: atazanavir; AZT: zidovudine; DTG: Dolutegravir; EFV: efavirenz; LPV: lopinavir; NVP: nevirapine; TDF: tenofovir disoproxil fumarate; OR: odds ratio; TDF: tenofovir disoproxil fumarate. CI: confidence interval. ART: antiretroviral therapy. For the multivariate analysis, the model was adjusted for age, NRTI backbone and anchor drug. P-values in boldface indicates those that were significantly associated ($p < 0.05$) with virological suppression.

Supplementary Table S3. Factors associated with virological suppression among young adults.

Factors	Regression model			
	**Crude OR (95% CI)**	**p-value**	**Adjusted OR (95% CI)**	**p-value**
**Sex**				
Males	0.920 (0.732-1.156)	0.474	0.966 (0.766-1.219)	0.772
Females	1			
**Treatment duration, month**				
6	0.894 (0.716-1.115)	0.319	0.857 (0.684-1.073)	0.179
12	1.205 (0.944-1.538)	0.135	1.149 (0.898-1.472)	0.269
24	**1.477 (1.099-1.984)**	**0.010**	**1.419 (1.055-1.910)**	**0.021**
36	1.409 (0.876-2.267)	0.157	1.356 (0.841-2.185)	0.211
>36	1			
ART regimen line, n (%)				
1st line	1			
2nd line	0.483 (0.282-0.827)	0.008		
3rd line	0.153 (0.031-0.758)	0.022		
**Backbone**				
TDF/3TC	1		1	
ABC/3TC	0.651 (0.245-1.734)	0.391	0.945 (0.337-2.650)	0.914
AZT/3TC	0.388 (0.075-2.003)	0.258	0.780 (0.140-4.357)	0.777
**Anchor drug**				
DTG	1		1	
EFV/NVP	0.904 (0.711-1.149)	0.409	0.936 (0.734-1.194)	0.596
**ATV/r/LPV/r/DRV/r**	**0.415 (0.249-0.689)**	**0.001**	**0.440 (0.252-0.769)**	**0.004**

3TC: Lamivudine; ABC: abacavir; ART: antiretroviral treatment; ATV: atazanavir; AZT: zidovudine; DTG: Dolutegravir; EFV: efavirenz; LPV: lopinavir; NVP: nevirapine; TDF: tenofovir disoproxil fumarate; OR: odds ratio; TDF: tenofovir disoproxil fumarate. CI: confidence interval. ART: antiretroviral therapy. For the multivariate analysis, the model was adjusted for sex, treatment duration, NRTI backbone and anchor drug. P-values in boldface indicates
